# Layer-by-Layer Deposited Multi-Modal PDAC/rGO Composite-Based Sensors

**DOI:** 10.3390/foods12020268

**Published:** 2023-01-06

**Authors:** Ammar Al-Hamry, Tianqi Lu, Jing Bai, Anurag Adiraju, Tharun K. Ega, Igor A. Pašti, Olfa Kanoun

**Affiliations:** 1 Measurement and Sensor Technology, Department of Electrical Engineering and Information Technology, Chemnitz University of Technology, 09107 Chemnitz, Germany; 2 University of Belgrade—Faculty of Physical Chemistry, Studentski trg 12-16, 11158 Belgrade, Serbia

**Keywords:** multi-modal sensor, poly(diallyl dimethyl ammonium chloride), reduced graphene oxide, layer-by-layer deposition, temperature sensor, relative humidity sensor, volatile organic compounds, electrochemical senor

## Abstract

Different environmental parameters, such as temperature and humidity, aggravate food spoilage, and different volatile organic compounds (VOCs) are released based on the extent of spoilage. In addition, a lack of efficient monitoring of the dosage of pesticides leads to crop failure. This could lead to the loss of food resources and food production with harmful contaminants and a short lifetime. For this reason, precise monitoring of different environmental parameters and contaminations during food processing and storage is a key factor for maintaining its safety and nutritional value. Thus, developing reliable, efficient, cost-effective sensor devices for these purposes is of utmost importance. This paper shows that Poly-(diallyl-dimethyl ammonium chloride)/reduced Graphene oxide (PDAC/rGO) films produced by a simple Layer-by-Layer deposition can be effectively used to monitor temperature, relative humidity, and the presence of volatile organic compounds as indicators for spoilage odors. At the same time, they show potential for electrochemical detection of organophosphate pesticide dimethoate. By monitoring the resistance/impedance changes during temperature and relative humidity variations or upon the exposure of PDAC/rGO films to methanol, good linear responses were obtained in the temperature range of 10–100 °C, 15–95% relative humidity, and 35 ppm–55 ppm of methanol. Moreover, linearity in the electrochemical detection of dimethoate is shown for the concentrations in the order of 10^2^ µmol dm^−3^. The analytical response to different external stimuli and analytes depends on the number of layers deposited, affecting sensors’ sensitivity, response and recovery time, and long-term stability. The presented results could serve as a starting point for developing advanced multi-modal sensors and sensor arrays with high potential for analytical applications in food safety and quality monitoring.

## 1. Introduction

Efficient food processing, manufacturing, and monitoring processes are some of the essential strategies in battling the worldwide rising famine epidemy, especially during the current economic crisis that also hit the food market. Based on statistical data, 11.3% of the World’s population is hit by famine, with a quarter of this number being in sub-Saharan Africa and over 500 million people in Asia [1]. In order to overcome this challenge effectively, it is necessary to implement monitoring rules for food production and processing in order to reduce the loss of valuable resources. Specifically, while many different, mutually interconnected factors, such as politics, religion, poverty, and inequality, contribute to global famine, significant amounts of food are lost in the post-harvesting and post-production period (as discussed further below), and these resources could be redirected to famine-affected regions. Another vital problem that impacts healthy food production is growing pollution, where many toxic substances such as pesticides end up in the final products, risking in that way human health.

Temperature and odor control in food processing is one of the key aspects during production, as any abnormal values of both parameters are strongly related to microbial growth and the quality of the final products [2,3]. In fact, there are pieces of evidence that in developed countries, nearly 30% of food is lost in the post-harvesting period [4]. Furthermore, as storage and processing conditions can influence food quality, it is also very important to consider monitoring methods, which could be performed by analyzing volatile organic compounds (VOCs) emitted, alcohols being the most common [5]. However, all these aspects are mutually connected as temperature and moisture affect the growth of microorganisms, which in turn cause food decomposition and emission of VOCs. Finally, monitoring pollutant residues in food, pesticides being of the major concern, is of crucial importance as they impose direct health risks [6].

In this regard, developing novel, cost-effective, and efficient sensors for monitoring environmental conditions in food processing and storage is essential, as well as the development of sensing platforms for monitoring different pollutants in food. For this reason, the research in sensing technologies is very active. For example, different solutions have been found, such as temperature sensors [7], humidity sensors [8], VOCs sensors [9,10,11], metal ions [12,13], as well as for pesticides and other contaminants [14,15]. In all these applications, sensitive materials based on conductive polymers and carbon materials play very important roles as they are cheap, affordable, and environmentally benign. Nevertheless, for any of the desired applications, a sensor has to fulfill several requirements, including good linearity, high sensitivity, low hysteresis, rapid response and recovery time, and selectivity.

Composites of carbon materials and conductive polymers have found their place in sensor technologies. Additional impetus for such composite-based sensors was the discovery of novel low-dimensional carbon nanostructures, primarily graphene, known for many exceptional properties and different applications [16,17,18,19]. Another important aspect is the development of novel sensor production strategies so that materials with controllable properties, supramolecular structure, and miniaturization can be achieved, such as different printing and deposition techniques, including Layer-by-Layer (LbL) deposition techniques [20,21]. There are many examples of various LbL-based sensors in the literature. The process is based on the self-assembly of oppositely charged layers, like carboxylated single-walled carbon nanotube (SWNT) self-assembly with a polycation, poly(diallyl dimethyl ammonium chloride (PDDA) used for pH sensing [22]. As polycations, different conductive polymers can be used, like polyaniline (PANI) [23,24], poly(dimethyldiallyl ammonium chloride) (PDDAC) [25], polyethylene glycol (PEG) [26], and as carbon material graphene oxide (GO) [27], CVD graphene [28], different nanotubular carbon forms [22,26], and others. These sensors have been applied for temperature sensing [29], humidity measurements [25,30], VOCs sensing [26,31], and also as electrochemical sensors [32]. In addition, these carbon-based nanomaterials are commonly demonstrated as multi-modal sensitive materials [33]. 

Different polymer/carbon combinations can be utilized for measuring different parameters, but the question is whether it is possible to reach composite formulations with more than one sensor application. This issue is highly important for rationalizing and economizing sensor production while not compromising on the sensors’ performances and applicability. Based on the above discussion, GO is a good candidate for different sensor applications with facile methods of preparation and coating. With the LbL technique, tuned film thickness, sensitivity, stability, and adhesion can be obtained [31,34]. The main aim of this research is to investigate and develop sensors as a proof of concept to monitor different environmental parameters, gases, and pesticides, which can be used for efficient food monitoring during processing, storage, and other applications. Here we present LbL-manufactured PDAC/rGO composite-based sensors, which can be used as temperature, relative humidity, and VOC sensors, while they also show potential for use in electrochemical sensors of pesticides. Sensors’ properties are affected by the number of deposited PDAC/rGO layers and temperature treatment, and, depending on the selected application, the sensor architecture can additionally be optimized to obtain the best possible sensor performance. The sensor properties of PDAC/rGO layers were investigated for their response to; temperature in the range of 10–100 °C, relative humidity (15–95%), methanol VOC (25–55 ppm), and dimethoate pesticide (100–700 µM/dm^3^). Optical properties were investigated by Raman spectroscopy, atomic force spectroscopy, and scanning electron microscopy. PDAC/rGO sensors were demonstrated to differentiate the changes in food and beverage.

## 2. Materials and Methods

### 2.1. Materials Preparation and Sensor Fabrication

GO oxide solution (0.4 wt.%) was purchased from Graphenea (San Sebastián, Spain) and used without further purification. For the LbL preparation of the sensor electrodes, it was diluted using high-purity deionized water to 0.1 wt.%. The PDAC used in this work was purchased from Sigma-Aldrich (Taufkirchen, Germany). It is a light-yellow viscous liquid with a solution concentration of 20 wt.%. For the LbL procedure, it was diluted to the concentration of 1 wt.% and used as such for all the experiments described from now on. 

Sensor electrodes were prepared using the LbL procedure, employing screen-printed silver interdigitated electrode (IDE) on Kapton HN substrate, with dimensions of 15 × 4 mm (see Figure S1a). Before the LbL process, the IDEs were cleaned with isopropanol and dried with nitrogen to remove dust contamination on the electrode film. To coat only the active area on the IDE electrodes, the undesired area around the silver IDE section, and on the back of the film completely using Oramask Film 810, purchased from Orafol GmbH, Oranienburg, Germany. Next, the silver IDE was fixed on the NEMESYS pump precision device (Cetoni GmBH, Korbußen, Germany), and the electrode was immersed in the PDAC solution for 5 min. After this step, IDE was rinsed in deionized water for 30 s, then taken out and slowly dried with nitrogen gas. In the next step, IDE was immersed in the GO solution for 5 min, after which the rinsing and drying step was repeated identically as previously described [35]. This way, a single bi-layer is formed, and the electrode is marked as (PDAC/GO)_1_. The above process is repeated *N* times, and such electrodes are designated as (PDAC/GO)*_N_*. Thus, *N* gives the number of deposited PDAC/GO bi-layers. The LbL process is schematically presented in Figure S1b.

After the LbL procedure, the (PDAC/GO)*_N_* electrodes were thermally reduced on a hot plate to obtain PDAC/rGO-*N*L sensor electrodes. The temperature was chosen by thermal reduction of the (PDAC/GO)_4_ electrode. The resistance of the obtained PDAC/rGO-4L sensor electrode reached a plateau after 200 °C, so temperature of 200 °C was subsequently used to reduce all the sensors reported here.

### 2.2. Sensor Physical and Chemical Characterization

Sensor electrodes were characterized using Scanning Electron Microscopy with Energy-Dispersive X-ray Spectroscopy (SEM-EDX), Raman spectroscopy, and Atomic Force Microscopy (AFM). 

Raman spectra, excited with a diode-pumped solid-state high brightness laser (excitation wavelength 532 nm), were collected on a DXR Raman microscope (Thermo Scientific, Waltham, MA, USA) equipped with an Olympus optical microscope and a CCD detector. The laser beam was focused on the sample using an objective magnification of 10×. The scattered light was analyzed by the spectrograph with a 900 lines mm^−1^ grating. Laser power on the sample was kept at 1 mW to prevent thermal degradation of the samples. The AFM analysis was performed using Agilent 5600LS (Keysight, Santa Rosa, CA, USA) in tapping mode. SEM-EDX characterization was performed using a Phenom ProX (Phenom, Eindhoven, the Netherlands).

### 2.3. Sensor Performance

#### 2.3.1. Temperature Measurement

For the static temperature measurements, a fabricated sensor was placed on the heating plate. To prevent uneven or incomplete sensor heating due to the upward bending of the sensor material, a Kapton film is placed over the sensor, followed by a metal plate to ensure that the sensor is in full contact with the heating plate. Sensor resistance was measured using Keithley Sourcemeter 2602. 

A preheated silicon oil tank (thermostat C12 CS, Lauda, Lauda-Königshofen, Germany) was used to determine sensor response time accurately. The sensors were fixed to a metal bracket controlled by an air pump and connected to the Keysight Sourcemeter. When the oil temperature is stable at 80 °C, the air pump pneumatic plunge arm inserts the sensor into the oil for an accurate response time measurement. The sensors were removed from the oil two minutes later, and the recovery time was measured.

#### 2.3.2. Humidity Measurement 

The relative humidity (RH) measurement system was composed of 5 parts: a computer with a LabVIEW program, measurement device, gas control system, Arduino UNO board with SHT85 RH-Temperature sensor, and test chamber (see Figure S2). The reference sensor was used to measure and control the actual humidity in the chamber. In this experiment, Agilent LCR Meter 4284A was used as the measuring device. In addition, two FLOW BUS (Bronkhorst, Veenendaal, the Netherlands) were used for the gas control system. The block and connection diagram is shown in Figure S2.

#### 2.3.3. VOCs Measurement 

Owlstone V-OVG (Owlstone, Connecticut, USA) was used as a gas-generating system. The VOCs gas (methanol) was mixed with nitrogen and introduced into the chamber containing the sensor electrode. The sensor response was measured using Keysight DAQ973A and processed using LabVIEW 2021 software. 

#### 2.3.4. Electrochemical Measurement

Electrochemical measurements were carried out in an all-glass one-compartment electrochemical cell. As working electrodes, modified Ag electrodes were used, while Saturated Calomel Electrode (SCE) and a wide Pt foil were used as a reference and counter electrode, respectively. As a supporting electrolyte 1 mol/dm^3^ KNO_3_ was used. Experiments were performed using Gamry Interface 1010E Potentiostat/Galvanostat/ZRA (Gamry instrument, Warminster, PA, USA). Measurements were performed in a quiescent solution. Cyclic voltammetry was investigated in the potential range −0.30 to +0.40 V vs. SCE. Working electrodes were produced by LbL procedure as described, but on continuous screen-printed Ag strips so that the performance of (PDAC/rGO-*N*L)@Ag is measured. In this case, Ag served as a substrate and a current collector. Analyte, organophosphate pesticide dimethoate, was injected stepwise in the electrolyte, and the voltammetric response of the sensor electrodes was detected.

## 3. Results and Discussion

### 3.1. Sensor Physical Properties 

SEM-EDX analysis was performed in order to confirm the deposition of GO and PDAC on the silver IDEs. As we reported previously [35], high transparency and low concentration of the GO sheets and PDAC prevented direct observation of deposited layers on the silver substrate. However, the EDX analysis confirmed the presence of carbon and oxygen (in the ratio 2:1), nitrogen which is present in PDAC, and the underlying Ag. This clearly confirms an effective functionalization of the silver electrode by PDAC/GO layers. Nevertheless, more direct pieces of evidence of the presence of GO and PDAC in the as-deposited films come from Raman spectroscopy. Based on the Raman spectra taken for the composites reduced at 100 °C it can be seen that some inhomogeneity is present for one and two layers, which disappear already for 4 L. Raman spectra clearly show the D and G bands of graphene, while for the samples with one and two layers, the characteristic bands of PDAC are also visible (see Figure S3). 

**Figure 1:**
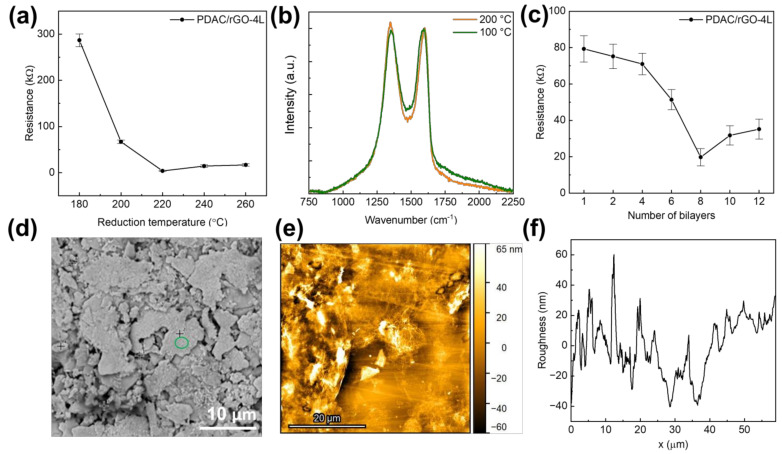
(**a**) Initial resistance of the proposed PDAC/rGO-4L sensors as a function of reduction temperature; (**b**) Raman spectra of PDAC/rGO-4L composite electrodes reduced at 100 °C and 200 °C (spectra were normalized so that the intensity of the G band is set to 1); (**c**) Initial resistance of the proposed PDAC/rGO sensors as a function of the number of deposited bi-layers; (**d**) Low-magnification SEM image showing overall morphology of the sensor; **(e)** AFM image showing overall topology of PDAC/rGO-2L sensor; (**f**) Roughness profile of the PDAC/rGO-2L sensor from AFM measurements.

Following the reduction of produced (PDAC/GO)*_N_* composites, the sensors’ resistance decreased as the reduction temperature was raised to 200 °C, after which a slight increase was seen (Figure 1a). The initial resistance of (PDAC/GO)_4_ is 286.9 kΩ. The initial resistance decreases rapidly when the reduction temperature is higher than 180 °C. As the reduction temperature rises to 220 °C, the initial resistance was found to be 3.8 kΩ for the PDAC/rGO-4L sensor. When the reduction temperature continued to rise, the initial resistance increased slightly. For this reason, sensors described starting in Section 3.2 have been produced with a reduction temperature of 200 °C. The primary effect of reduction temperature on film resistance is sought in removing oxygen-containing functional groups from GO. The oxygen-containing functional groups break the ultra-long distance conjugated large π-bonds on the graphene surface, reducing the electron migration rate. The removal of oxygen-containing functional groups, although generating defects, caused the electron migration rate to rebound [10,36]. The formation of defects during the reduction was confirmed here using Raman spectroscopy. For the PDAC/rGO-4L sensor, it is clear that the intensity of the D band, associated with the presence of defects [37], increases when the composite is reduced at 200 °C (see Figure 1b and Figure S3).

When the number of film layers is less than or equal to four layers, although the initial resistance of the sensor decreases with the increase of the number of layers, the decrease is not large. The initial resistance of the sensor decreases from 79.3 kΩ at one layer to 71 kΩ at four layers, a decrease of approximately 10%. When the number of film layers exceeds four, the initial resistance begins to drop significantly. When the number of layers is eight, the initial resistance of the sensor reaches the lowest value, 19.8 kΩ. Starting at that point, as the number of layers of the sensor continues to rise, the initial resistance of the sensor begins to rise again. The increase in the film conductivity with the rising number of layers is a consequence of the increasing number of channels across the layers, causing an increase in electron mobility. Sarker and Hong [38] reported a similar result: the sheet resistances of the multilayer films decreased exponentially as the number of bi-layers increased, reaching a minimum resistance for 15 bilayers.

**Figure 2:**
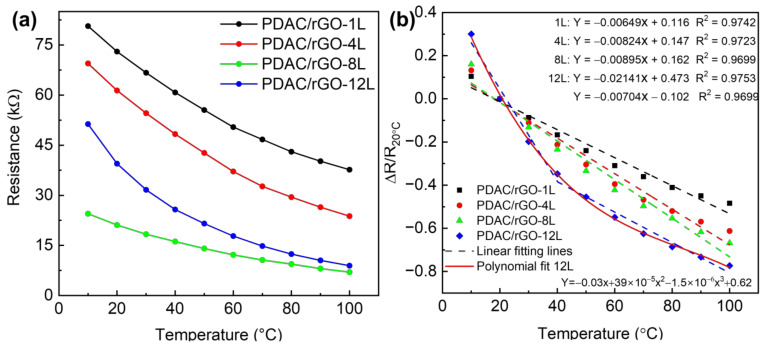
Static thermal properties of PDAC/rGO sensors: (**a**) Resistance of PDAC/rGO sensors and (**b**) sensitivity of PDAC/rGO.

The SEM analysis was performed on the Ag part of the interdigitated structure covered by PDAC/rGO layers to ensure good conductivity of the analysis spot (see Figure 1d). It was only possible to observe the overall morphology with Ag flakes, but not PDAC/rGO composite, while the EDX analysis indicated the presence of the bi-layers. This suggests very thin deposits are formed, which was confirmed by the AFM analysis (see Figure 1e and Figure 2f). It was found that the thickness of the composite in the case of the PDAC/rGO-2L sensor is very low. However, it can be assumed that as the number of layers with PDAC/rGO gradually increases, the film formation becomes more and more effective. On the other hand, the roughness of the PDAC/rGO-12L sensor composite film is still more than twice that of PDAC/rGO-2L. The RMS roughness of PDAC/rGO-2L was found to be 30.87 nm, and that of PDAC/rGO-12L was 64.27 nm.

### 3.2. PDAC/rGO-Composites for Temperature Measurements

First, the response and quantitative relationship between the number of sensor layers and temperature response was established. Figure 2a shows the response curves of 1-layer, 4-layer, 8-layer, and 12-layer sensors to temperature changes in the range of 10 °C–100 °C. The resistance value at room temperature, i.e., 20 °C, was chosen as the initial resistance value (R_20 °C_) so that the response is calculated as ΔR/R_20 °C_. The calibration curves of the PDAC/rGO sensors are shown in Figure 2b. The sensitivity of the PDAC/rGO-1L, -4L, and -8L were 48.43%, 61.26%, and 66.80% at 100 °C, respectively. The sensitivity of the PDAC/rGO-12L sensor increased from 19.83% at 30 °C to 77.31% at 100 °C. This is because, as the number of sensor layers increases, the rGO surface can ionize more electrons from more oxygen-containing functional groups, leading to an increase in sensor sensitivity. The sensitivity curve for the different numbers of bi-layers is provided in Figure S5a. The temperature coefficient of resistance (TCR = ΔR/R_0_ 1/ΔT), as extracted from the linear fitting, are −0.65, −0.85, and −0.89%/°C for PDAC/rGO-1L, -4L, and -8L, respectively. For PDAC/rGO-12L, it is −2.1%/°C in the range (10–40 °C) and −0.7%/°C in the range (40–100 °C). 

**Figure 3:**
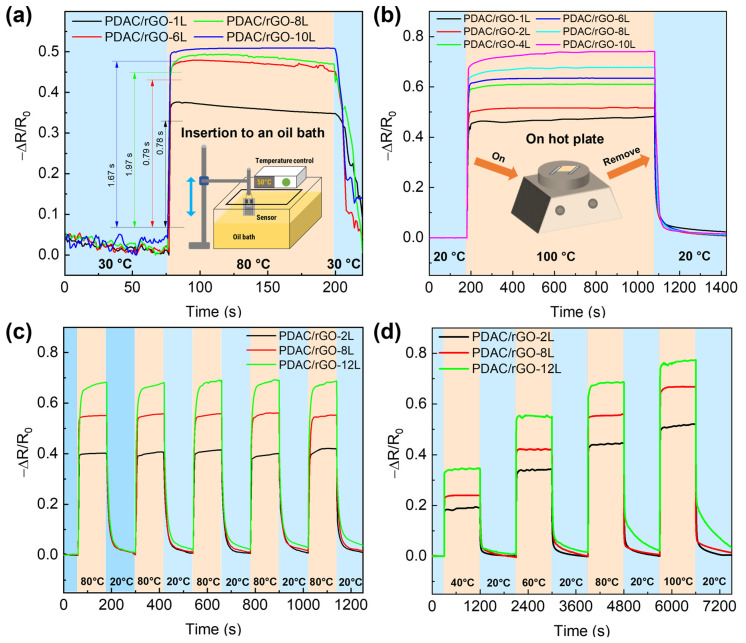
Dynamic thermal properties of PDAC/rGO sensors: (**a**) Response curves of PDAC/rGO sensors plunged abruptly in hot oil bath, and (**b**) recovery curves of PDAC/rGO sensors for different numbers of layers removed suddenly from hot plate; (**c**) repeatability and (**d**) step curves of thermal properties of PDAC/rGO sensors upon exposure to different heating–cooling programs.

In the next step, the response and recovery time of the sensors are addressed. Figure 3a shows the dynamic thermal properties of PDAC/rGO sensors in the oil tank preheated to 80 °C, and Figure S4b gives the response time curve of PDAC/rGO sensors. The PDAC/rGO sensor response time increases as the number of layers increases. When the number of sensor layers is less than 6, the temperature response time is less than 1 s (0.78 s and 0.79 s for the one-layer and eight bi-layer-sensor, respectively). When the number of sensor layers is more than six, the PDAC/rGO sensor’s response time is 2 s. To determine the recovery times, the sensors were heated from room temperature (20 °C) onto a hot plate to 100 °C. After 900 s of complete heating (when sensor response remained stable), the sensors were quickly removed from the hot plate and placed in a room-temperature environment, providing curves for determining the recovery time (see Figure 3b). The recovery time of PDAC/rGO sensors is about 24.87 s–35.80 s (see Figure S4c). 

In order to explore the response of different sensors to rapid temperature changes, the repeatability curve is measured. The measurements were carried out on a hot plate. The sensors were quickly placed on top of the hot plate at 80 °C and then at room temperature of 20 °C for 120 s only in each state for five consecutive sets of rapid cycling experiments. This protocol resulted in the repeatability curve shown in Figure 3c. PDAC/rGO-12L sensor was essentially equilibrated, and the 2L and 8L sensors were fully equilibrated. The recoverability of the 2L and 8L sensors also remains largely unchanged, but that of the 12L sensor decreases as the number of cycles increases. 

In the second set of experiments, successive stages of heating at different temperatures and recovery of the sensor are tested. In these experiments, 40 °C, 60 °C, 80 °C, and 100 °C are chosen as the test temperatures. First, the sensor was placed on a hot plate from room temperature (20 °C) to the lowest test temperature of 40 °C and subjected to temperature response measurements for 900 s. After 900 s, the sensor is moved to room temperature for a recovery test for 900 s. The step curve is then repeated by placing the sensor on the hot plate at 60 °C, 80 °C, and 100 °C to obtain the step curve of the sensor, as shown in Figure 3d.

The calibration curves of PDAC/rGO sensors to temperature changes in these two sets of experiments were consistent. The PDAC/rGO sensors with the same number of layers have practically the same response time to temperature and basically do not change with the temperature. Further, the recovery time of 2L and 8L PDAC/rGO sensors to temperature remains almost unchanged, but the recoverability is slightly reduced. The PDAC/rGO-12L sensor has a significant downward trend in temperature measurement recovery time and recoverability due to the slow cooling process and heat capacity of the substrate.

The long-term stability measurements were performed by assessing sensor sensitivity at 40 °C, 60 °C, 80 °C, and 100 °C. The measurement interval was five days, and seven measurements were taken in total over one month under the same ambient conditions. Over one month period, the long-term stability of the PDAC/rGO-8L sensor decreased slightly (see Figure S4d). At the start date, the sensitivities of the PDAC/rGO sensor at 40 °C and 100 °C are 23.98% and 66.76%, respectively. By day 30 was reached, the sensitivity dropped to 23.4% at 40 °C and 64.44% at 100 °C. At 40 °C, the sensitivities of the PDAC/rGO sensor decrease by 0.27 and 0.58 at 15 and 30 days, respectively. When the test temperature reaches 100 °C, the sensitivities decrease by 1.12% on day 15 and 2.32% on day 30. Based on the obtained results, it can be safely concluded that the stability of the PDAC/rGO sensor is excellent.

### 3.3. PDAC/rGO-Composites for Relative Humidity Measurements 

**Figure 4:**
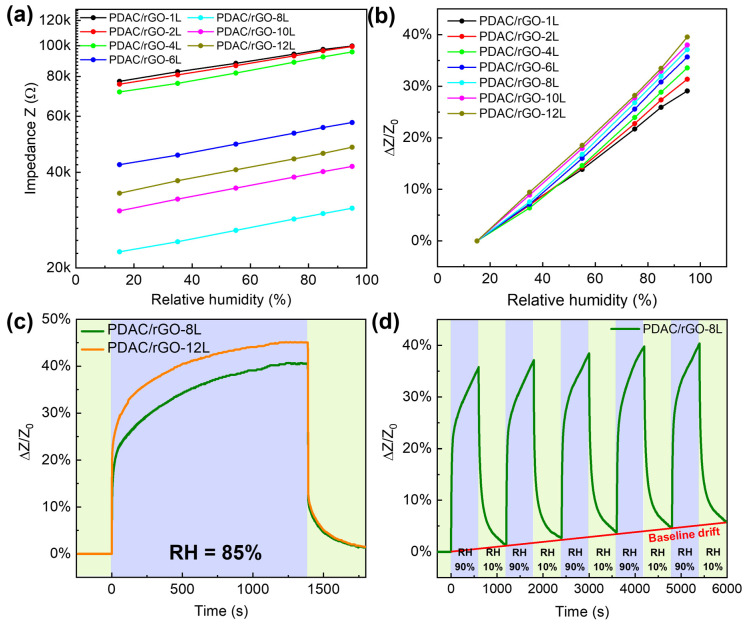
Static and dynamic characteristics of PDAC/rGO humidity sensors: (**a**) Impedance of PDAC/rGO sensors; (**b**) sensitivity of PDAC/rGO sensors; (**c**) response curves of PDAC/rGO-8L and PDAC/rGO-12L sensors; (**d**) repeatability of the humidity characteristic of PDAC/rGO-8L sensor.

For the relative humidity measurements, the range of 15–95 RH% is investigated (see Figure 4a). The impedance measured in pure nitrogen gas was used to obtain the initial impedance value (Z_0_) for the construction of calibration curves. The impedance was measured for relative humidity in the chamber set to 15%, 35%, 55%, 75%, 85%, and 95%. The impedance values of the sensors gradually increased as the humidity rose. PDAC/rGO sensors showed good humidity sensor characteristics due to the properties of rGO, where water molecules penetrate the interlayer between rGO flakes, causing the increase of tunneling effect and, thus, a decrease in the conductivity. Even at high RH%, there was no formation of water film due to hydrophobicity of rGO [39]. Expansion of the polymer-rGO composite film may as well play a role caused by water adsorption. The effect is the opposite as the relative humidity decreases. Figure 4b shows the humidity calibration curve for PDAC/rGO-*N*L sensors. The sensor’s sensitivity increased monotonically with the number of layers (see Figure S5a, Supplementary Information) and reached a maximum of 39.56% PDAC/rGO-12L sensor at 95% RH. 

Once the calibration curves have been established, the humidity sensor is tested more deeply. Figure 4c presents the response curve of PDAC/rGO-8L and PDAC/rGO-12L sensors. In terms of recovery, PDAC/rGO sensors showed recovery times of around 120 s.

PDAC/rGO-8L was chosen to investigate the repeatability of the sensor. At the beginning of the measurement, the sensor was placed in a chamber at 10% RH, and when stabilized, the humidity of the environment chamber was changed to 90% RH. After 600 s, the relative humidity is changed back to 10%, and this process was repeated five times (see Figure 4d). An obvious drift in the sensor response was seen due to the incomplete removal of adsorbed water during the cycling.

Finally, the long-term stability curve of the humidity response of the sensors is assessed using the PDAC/rGO-8L sensor. The measurement intervals and experiment duration were the same as in the case of temperature measurements, and sensor sensitivity for 15%, 35%, 55%, 75%, and 95% relative humidity was checked over one month (see Figure S5b). At a relative humidity of 15%, the initial sensitivity of the sensor was 5.882%, which decreased by 0.47% on day 15 and by 0.912% on day 30. At a relative humidity of 95%, the initial sensitivity was 46.03% and dropped by 1.009% and 1.987% for day 15 and day 30, respectively. This undoubtedly indicates the good stability of the sensors at different humidity levels.

### 3.4. PDAC/rGO-Composites for VOCs Measurements

**Figure 5:**
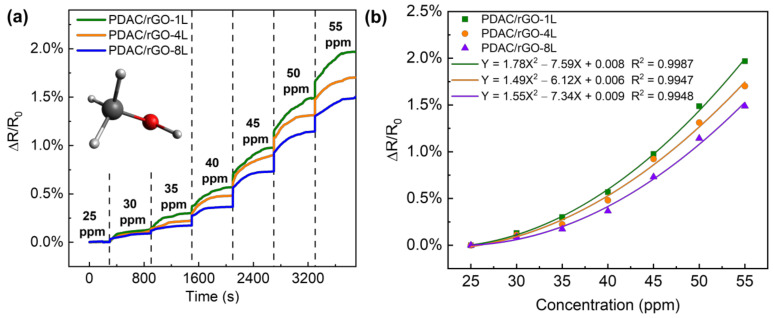
Response curve of the PDAC/rGO sensor to methanol gas (**a**) step response with time and (**b**) calibration curve.

The VOC response of the PDAC/rGO sensor is tested using the VOC generator as the VOC gas generator and methanol as the gas source, ranging from 25 ppm to 55 ppm. The sensor resistance value when the methanol gas concentration is 25 ppm is used as the initial resistance of the sensor (see Figure 5a). The response curve of the PDAC/rGO sensor to the methanol gas concentration and the calibration curves are shown in Figure 5b. Using the quadratic functions to link sensor response to the methanol concentration, it can be seen that sensitivity increases with methanol concentration.

The response of the PDAC/rGO sensor is positively correlated to the concentration of methanol. In contrast to the case of the relative humidity measurements, the highest sensitivity was observed for PDAC/rGO-1L sensor (1.97%) and the lowest for PDAC/rGO-8L, so the sensitivity dropped as the number of bi-layers increased. When the methanol concentration was below 40 ppm, the sensitivity changes for all three layers of PDAC/rGO sensors gradually increased and were very unstable, and when the methanol concentration was higher than 40 ppm, the change in sensitivity for all three sensors remained rather stable. After the methanol concentration exceeded 40 ppm, the sensitivity change of the three sensors was basically linear with the concentration change. For each 5 ppm increase in the methanol concentration, the sensitivity of the PDAC/rGO-1L, PDAC/rGO-4L, and PDAC/rGO-8L increases by approximately 0.5%, 0.4%, and 0.35%, respectively. Since a larger range of gas concentrations can be allowed by the diffusion tube in the VOC generator, the sample flow can also be expanded to a larger value so that the gas in the chamber can also quickly reach the measured concentration when the higher gas concentrations are required. The response times of the sensors, therefore, remain essentially the same.

### 3.5. PDAC/rGO-Composites as Electrochemical Sensors for Organophosphate Pesticides

**Figure 6:**
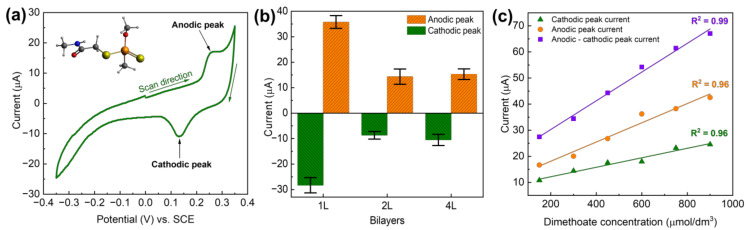
(**a**) Cyclic voltammetry of dimethoate at (PDAC/rGO-1L)@Ag, dimethoate concentration 100 µmol dm^−3^, inset shows the molecular structure of dimethoate; (**b**) voltammetric response of (PDAC/rGO-*N*L)@Ag electrodes for dimethoate as a function of the number of layers, dimethoate concentration of 200 µmol dm^−3^; (**c**) response of the (PDAC/rGO-1L)@Ag electrode in as the function of dimethoate concentration.

In addition to using LbL-produced PDAC/rGO layers for temperature monitoring, relative humidity, and VOC measurements, here we also briefly communicate that the same fabrication approach can be used for the electrochemical detection of pesticides. While the method is not fully optimized, the proof-of-concept is clear and provides added value to the multi-modal PDAC/rGO-based sensors. As shown previously [40], the electrochemistry of dimethoate at (PDAC/rGO-*N*L)@Ag is interesting, showing clear anodic and cathodic peak corresponding to dimethoate oxidation and reduction of the oxidation products (see Figure 6a). Depending on the number of the deposited bi-layers, the response is different, and for 1, 2, and 4 bi-layers, we found that the highest response is for the (PDAC/rGO-1L)@Ag electrode (see Figure 6b). When cyclic voltammograms are recorded, linearity in the response, taken as anodic or cathodic peak current versus concentration of dimethoate, was confirmed (see Figure 6c). The highest sensitivity and the best linearity were observed when the difference between anodic and cathodic peak current was plotted as the function of dimethoate concentrations. While the sensor is not fully optimized for the electrochemical detection of dimethoate, and the linearity range is, to this point, confirmed for relatively high dimethoate concentrations (order of 10^2^ µmol dm^−3^), it is clear that PDAC/rGO composite layers can also be used for electrochemical applications. 

### 3.6. Critical Evaluation of Sensors’ Properties

Here we have demonstrated the multi-modal nature of the LbL-produced PDAC/rGO composite-based sensors for temperature monitoring, relative humidity measurements, VOCs measurements, and electrochemical detection of dimethoate. The behavior of produced sensors varies depending on the different operational conditions, while the number of deposited layers also has a noticeable impact on the materials’ performance. Considering temperature measurements, the resistance decreases with the increase in temperature and vice versa. Although the increase in temperature intensifies the irregular motion of molecules and causes the mobility of free electrons to drop slightly, the number of free electrons increases faster with the increase of temperature, so the resistance of material at high-temperature decreases. It was proved that the second effect becomes dominant when the temperature exceeds 120 °C. In combination with rGO, PDAC does not play a dominant role in response to temperature changes and only affects the initial conductivity, as reported in [41]. The temperature response dependence of the proposed sensor is not only determined by the nature of the insulating polymer but also by the intrinsic properties of the reduced graphene oxide-based component. Thermal treatment at 200 °C used here caused the reduction of a large number of oxygen-containing functional groups in parental GO. Thus, produced rGO exhibits a p-type semiconductor behavior (having a negative temperature coefficient) whose resistance decreases with increasing temperature, as reported in [42,43]. The mechanism can be explained as Arrhenius-like temperature dependence of resistance, indicating a band gap dominating transport behavior [44].

Opposite to the case of the increasing temperature, there is a positive response to the increasing humidity (increasing impedance). The resistance of the proposed sensor increases with humidity as the resistive response of the insulating polymer is related to the degree of moisture absorption of the material. Cavallo et al. [45] demonstrated that at an initial environment of high relative humidity (65–90%), the polymer swells due to the continuous absorption of water. This process increases the distance between its molecular chains, hindering the charge-hopping process and reducing the electrical conductivity. Moreover, the increase in the number of deposited bi-layers increases the sensitivity of the relative humidity measurements (see Figure S5a). Namely, as the number of sensor layers increases, the PDAC/rGO film absorbs more moisture and expands, therefore increasing the response. For the PDAC/rGO sensor, the more layers there are, the more active cations can bind to water and, therefore, the higher the sensitivity [46].

In the cases of both temperature and relative humidity measurements, the sensitivity decayed slightly over one month of monitoring. In the case of humidity measurements, the sensitivity decreases practically in a linear fashion, and the rate is essentially constant over time. The performance of the sensors decayed faster for higher relative humidity. The PDAC/rGO-8L sensor has shown the largest sensitivity drop of 1.987% (absolute value) at 95% relative humidity in a long-term stability test, so the humidity response stability can be considered excellent.

In the case of the VOCs (methanol) detection, a positive sensor response is seen, i.e., increasing resistance with methanol concentration. The effective response of PDAC/GO films to methanol is a combination of PDAC and rGO contributions. The mechanism of the response of rGO to VOC has been explained by filling the defects on the rGO surface, resulting in interrupted long-range charge transport and increased resistance [10]. We note that studied sensors responded well to rising methanol concentration, but it was not the case with acetone. Methanol is more likely to swell the PDAC/rGO sensor surface and expand the distance between adjacent rGO domains, thereby increasing sensitivity. The effectiveness of the response of PDAC to methanol and the low response to acetone was previously confirmed by Al-Hamry et al. [47], who tested the PDAC/rGO-8L sensor at 2000 ppm with a sensitivity of about 15%. It is rather interesting to note that the sensor with only one bi-layer is the most sensitive in the case of methanol detection. This is likely because methanol cannot penetrate through thicker LbL films that remain partially unaffected by its presence and thus buffer the sensor response. 

Finally, considering the electrochemical detection of dimethoate, the sensor behavior is rather promising, but it cannot compete yet with some state-of-the-art sensors based on aptamers or molecularly imprinted polymers [15,48,49]. However, we note that the first logical step towards improving the linear range and reducing the limit of detection is the application of more advanced electrochemical techniques, like square wave voltammetry or differential pulse voltammetry. On the other hand, it is also important to observe that the most efficient electrochemical sensor is the one with one deposited bi-layer. This might indicate that the electrochemical reaction occurs at the interface between Ag and the deposited bi-layers. For thicker layers, it is difficult for dimethoate, which is a much larger molecule compared to methanol and water (see Figure 6a, inset), to reach the interface and undergo electrochemical transformation. Another important point is to note that the electrodes for electrochemical testing cannot be reused. This result suggests that harsh electrochemical conditions cause irreversible changes in the electrode structure, while there is also a possibility that the reaction products remain on the electrode and block active sites for electrochemical reactions of dimethoate.

To put the presented results in a broader context, we compare the performance of the presented PDAC/rGO-*N*L sensors to those previously reported in the literature (see Table 1). Taking the multi-modal aspect of our proposed sensors into account, they stand hand to hand with sensors specifically tailored for different applications. 

**Figure 7:**
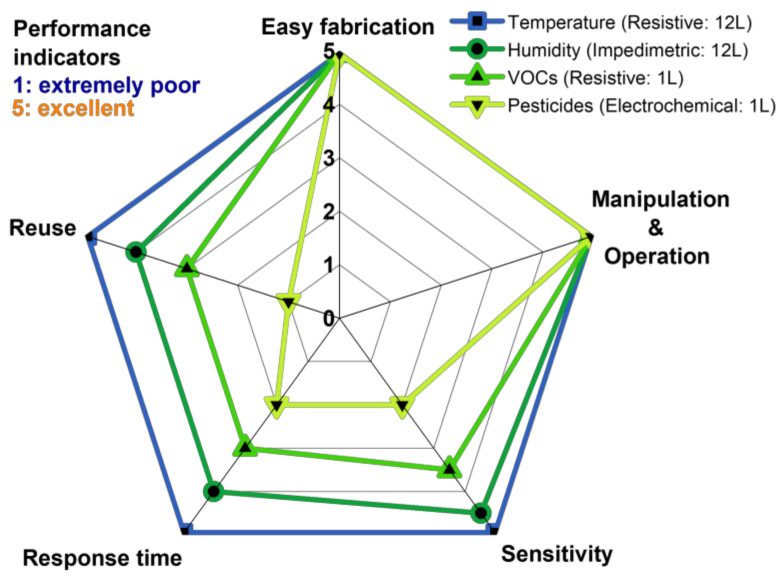
Spider diagram assessing the overall performance of PDAC/rGO-*N*L sensors for different applications presented in this work.

Based on the comparison with the literature data and the results provided in Section 3, there is certainly some space for improving the proposed sensors. However, a general evaluation of the performance reached to this point is presented in Figure 7. The temperature measurement performance of the presented sensors is appreciable, but in some other applications, further improvements are essential. For example, there is a significant drift in the humidity response characteristic, while the reuse for electrochemical applications and the corresponding sensitivity/response has to be significantly improved. 

**Figure 8:**
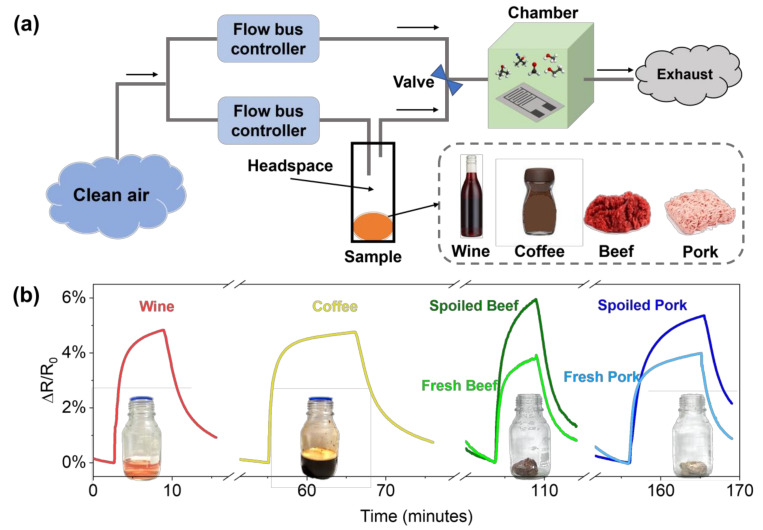
(**a**) Schematic representation of the total VOC measurement system for real samples; (**b**) the responses of the PDAC/rGO sensor to VOCs from beverage and meat samples.

Finally, to demonstrate the direct applicability of PDAC/rGO sensors in food monitoring, we show the overall response of the PDAC/rGO to VOCs from two beverage samples (wine and coffee) and two meat samples (grounded beef and pork) in Figure 8. The experiment was performed as presented in Figure 8a. The sample was placed in a glass container for 15 min to fill the headspace with volatile gas. The measurement chamber with the proposed sensor was first filled with a clean and dry airflow, and then the headspace of the sample container was directed through a switching valve. Both airflows were controlled by the flow bus controller. The information on the real tested samples is shown in Table S1. The sensor gave specific responses to each of the samples (see Figure 8b), which is not due to humidity, as the response to ground beef is the highest. Moreover, the response and recovery time are different for each sample, suggesting that the sensor is effectively responding to total VOCs from a specific sample. This study shows the ability of the sensor to be used in different cases for food quality monitoring which can be extended to give complex information about the food state and quality. Nevertheless, the calibration of sensors in different controlled environments needs to be performed and integrated with machine learning techniques so as to obtain an accurate and reliable fingerprint of food and beverages.

## 4. Conclusions

Taking the importance of precise monitoring of different environmental parameters and contaminants in food processing, developing sensitive and cost-effective sensors is of utmost importance. In this paper, we have shown that PDAC/rGO composites produced by an LbL procedure can be effectively used for temperature and relative humidity monitoring, VOC detection, and electrochemical measurements of organophosphate pesticide dimethoate. In the case of temperature monitoring, the proposed sensors show relatively short response times in the range of 0.78–1.6 s and recovery times in the range of 24–35 s, while the sensitivity increases with the number of bilayers deposited on the electrode. In the case of relative humidity measurement, the effect of the number of deposited bi-layers is similar, but by increasing the number of layers, the sensitivity changes are not significant. The long-term stability of the proposed sensors for temperature and relative humidity measurements is excellent, as the responses changed to a small extent over one month of testing. Further, the methanol gas sensitivity of the PDAC/rGO sensor decreases with the number of deposited PDAC/rGO bi-layers. Good linearity is observed for methanol concentrations above 35 ppm. The PDAC/rGO-1L sensor measured a maximum sensitivity of 1.97% at a methanol concentration of 55 ppm. PDAC/rGO-1L is also the most sensitive for the electrochemical detection of dimethoate, although further improvements in this direction are absolutely necessary. With the numerous applications of LbL self-assembly layers in the field of sensors, further advancements are expected, particularly toward developing multi-modal sensors and sensor arrays. Such multi-sensors could have a tremendous impact on monitoring food safety and quality, as demonstrated by sensing total VOCs from two beverages (coffee and wine) and meat samples (pork and beef). Because of their demonstrated capabilities, sensor arrays based on LbL-PDAC/rGO could be developed to detect different physical and chemical phenomena to serve as an electronic nose or tongue where artificial intelligence can be utilized to extract desired information. 

## Figures and Tables

**Table 1:** The performance comparison of previously reported LbL-manufactured sensors and those reported in this work (RH—relative humidity). For this comparison, the sensors with carbon-based nanomaterials were considered.

Polymer	Nanomaterial	Target	Linear Range	Sensitivity	Response Time	Reference
PEDOT:PSS	Graphene	Temperature	33–45 °C	0.06 %/°C	20 s	[50]
Polyaniline	Graphene		25–80 °C	1.2 %/°C	-	[51]
PEDOT:PSS	CNT		30–80 °C	0.64 %/°C	4.8 s	[52]
PDAC	rGO		10–100 °C	0.7–2.1 %/°C	0.78 s	This work *, **
S-PANI	-	Humidity	50–90%	60% (90% RH)	15–27 s	[23]
PDDA	GO		11–97% RH	8.69–37.43%	108–147 s	[30]
PANI	GO		11–97% RH	20 Hz/% RH	5–13 s	[24]
PDDAC	GO		11–97% RH	25.4 Hz/% RH	1–7 s	[25]
PDAC	rGO		15–95% RH	46% (100% RH)	~10 s	This work *
PEG	MWCNT	VOCs	1–60 μM	-	-	[32]
PEG	MWCNT		10–1000 ppm	0.06 %/ppm	110 s	[26]
PDAC	rGO		35–55 ppm	0.1 %/ppm	<10 s	This work **

* 12 bi-layers; ** 1 bi-layer.

## Data Availability

The data are available upon request to the corresponding author.

## Supplementary Materials

The following supporting information can be downloaded at: https://www.mdpi.com/article/10.3390/foods12020268/s1, Figure S1. (a) Schematic representation of the screen-printed Ag electrode used for the LbL deposition of PDAC/GO bi-layers; (b) Protocol for the LbL deposition of the PDAC/GO bi-layers and (c) photograph of the LbL PDAC/GO-based sensor after thermal reduction; Figure S2. The block and connection diagram of the humidity measurement system; Figure S3. Raman spectra of PDAC/rGO composite electrodes reduced at 100 °C with (a) 1 bi-layer; (b) 2 bi-layers; (c) 4 bi-layers. Yellow and green spectra are taken at two different spots of the sample (marked Spot A and Spot B); Figure S4. Temperature measurement (a) sensitivity curve as the function of the number of the PDAC/rGO layers; (b) response time as the function of the number of the PDAC/rGO layers; (c) recovery time as the function of the number of the PDAC/rGO layers; (d) Long-term stability curves for the temperature measurements using PDAC/rGO-8L sensor; Figure S5. (a) Sensitivity of the response as response curve of different layers for the relative humidity measurements; (b) Long-term stability curves for the relative humidity measurements using PDAC/rGO-8L sensor; Table S1. Information of the detected real samples.
