# Supplementary material for: Layer-by-Layer Deposited Multi-Modal PDAC/rGO Composite-Based Sensors
Source: Foods. 2023 Jan 6;12(2):268. doi: 10.3390/foods12020268 (PMC9857774; doi:10.3390/foods12020268)
Supplement: Supplementary file 1 [file foods-12-00268-s001.zip › foods-2113822-supplementary.pdf]

foods

Supplementary materials

Layer-by-Layer Deposited Multi-Modal PDAC/rGO Composite-Based Sensors

Ammar Al-Hamry [1], Tianqi Lu [1], Jing Bai [1], Anurag Adiraju [1], Tharun K. Ega [1], Igor A. Pašti [2] and Olfa Kanoun [1,*]

[1] Measurement and Sensor Technology, Department of Electrical Engineering and Information Technology, Chemnitz University of Technology, 09107 Chemnitz, Germany
[2] University of Belgrade—Faculty of Physical Chemistry, Studentski trg 12-16, 11158 Belgrade, Serbia
* Correspondence: olfa.kanoun@etit.tu-chemnitz.de

Supplementary figures:

(a)

(b)

(c)

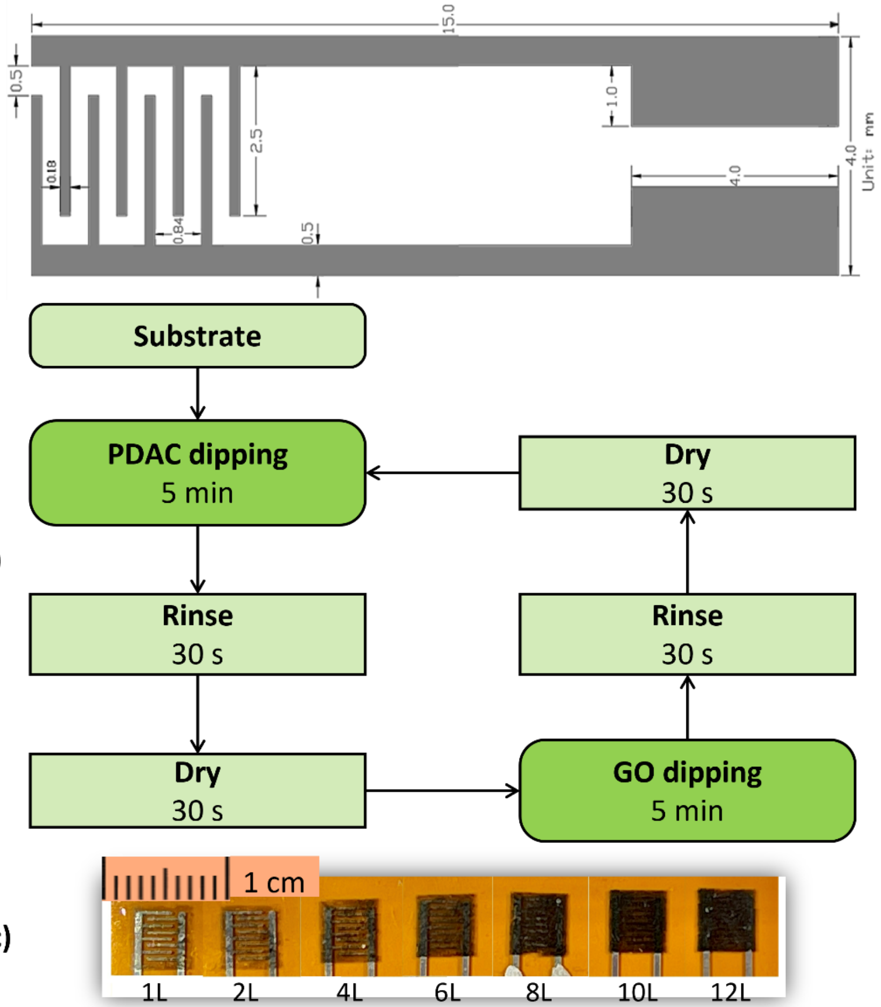

Figure S1. (a) Schematic representation of the screen printed Ag electrode used for the LbL deposition of PDAC/GO bi-layers; (b) Protocol for the LbL deposition of the PDAC/GO bi-layers and (c) photograph of the LbL PDAC/GO based sensor after thermal reduction.

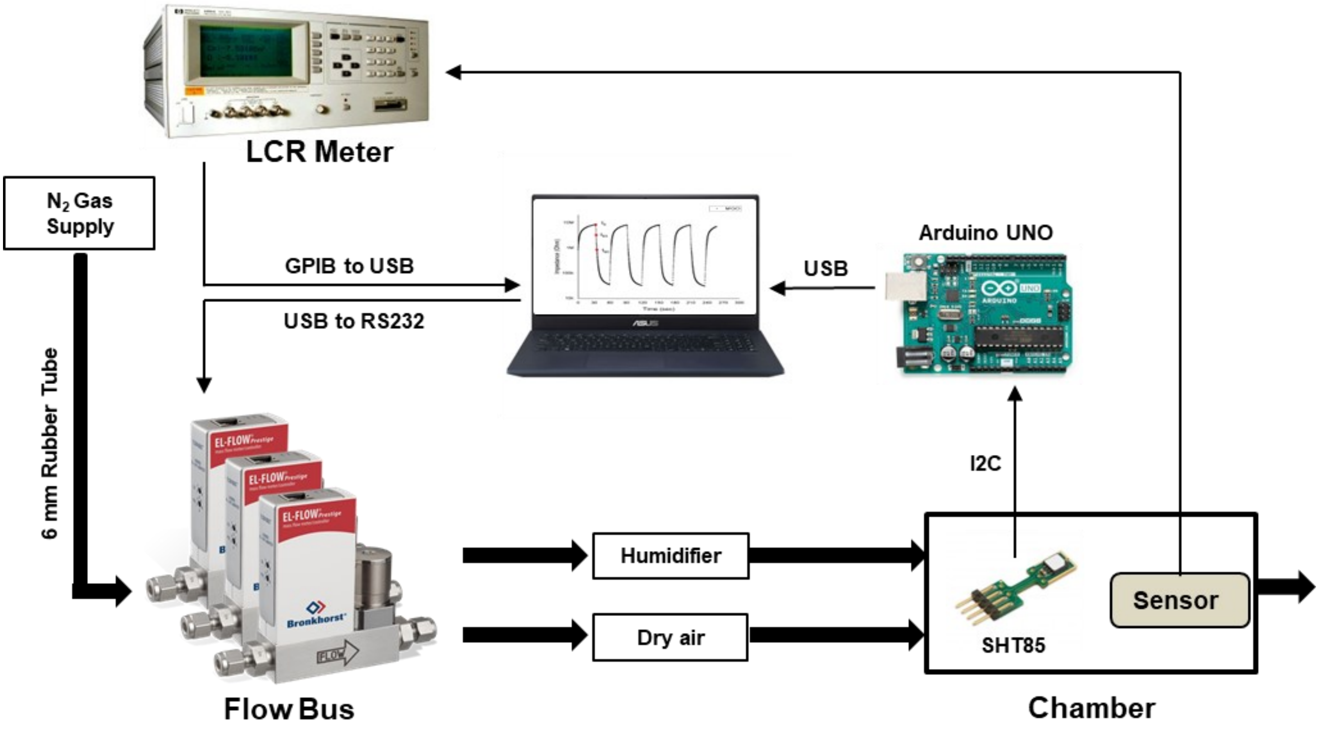

Figure S2. The block and connection diagram of the humidity measurement system.

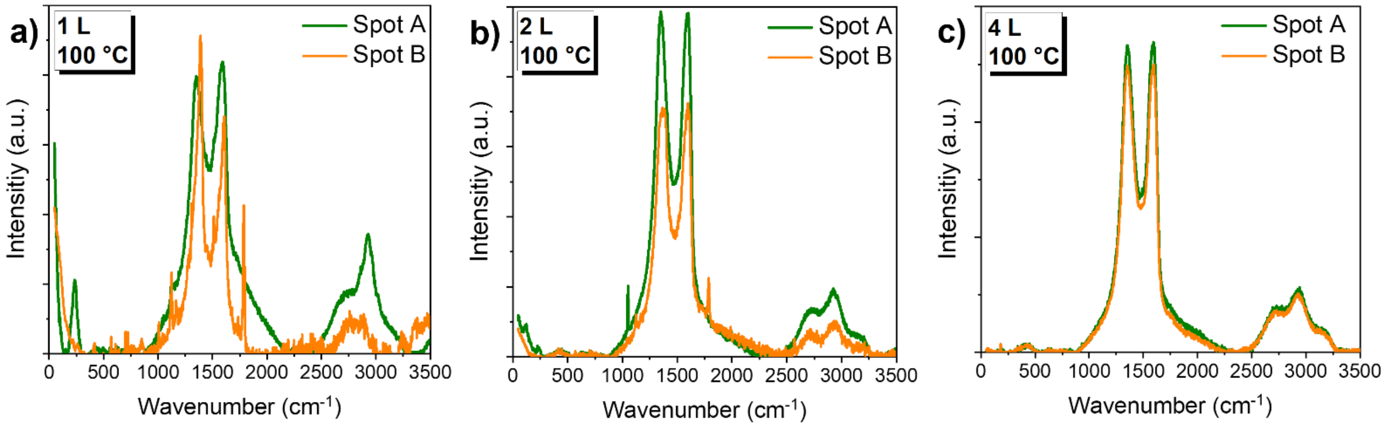

Figure S3. Raman spectra of PDAC/rGO composite electrodes reduced at 100 °C with (a) 1 bi-layer; (b) 2 bi-layers; (c) 4 bi-layers. Yellow and green spectra are taken at two different spots of the sample (marked Spot A and Spot B).

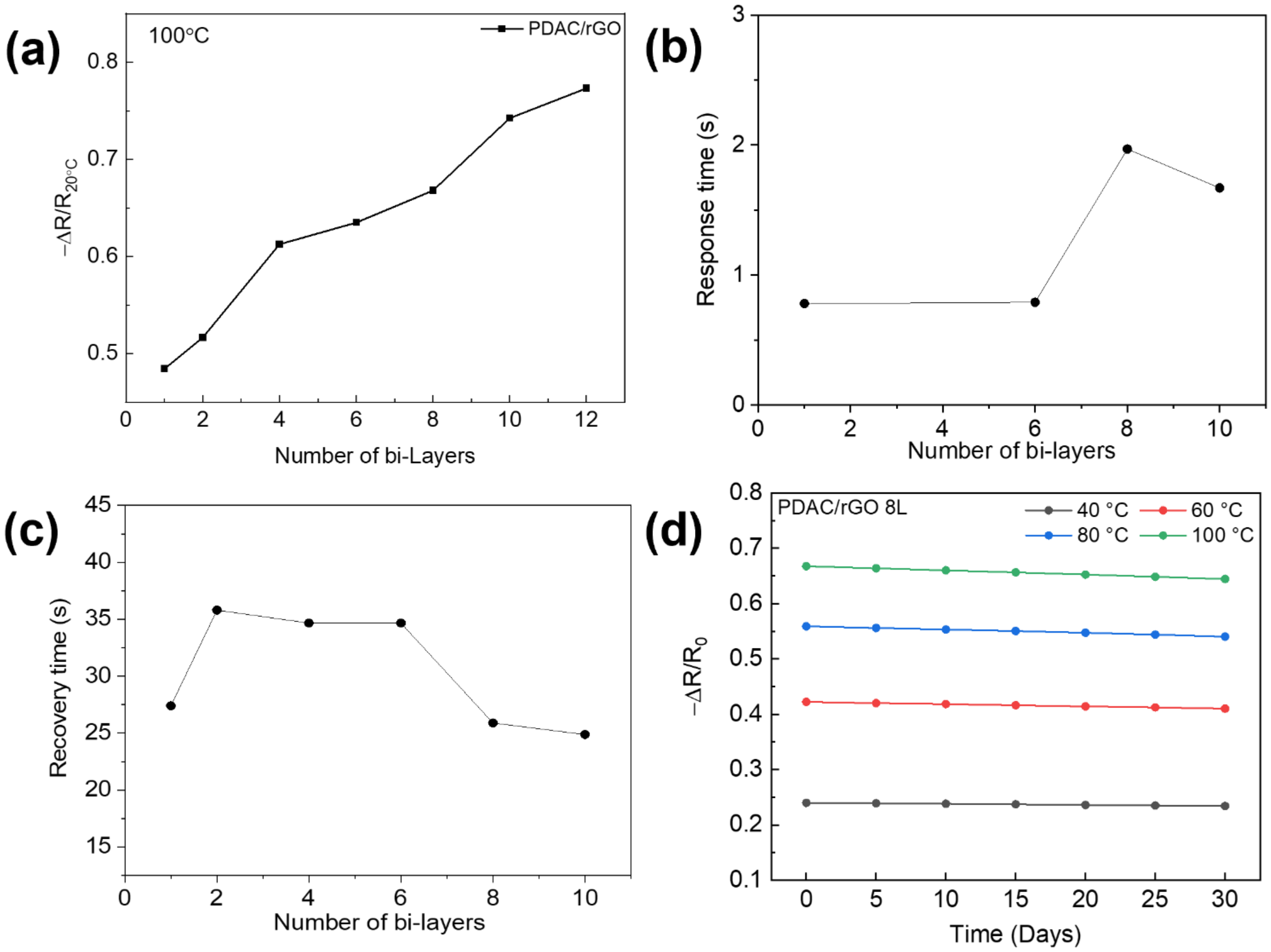

Figure S4. Temperature measurement (a) sensitivity curve as the function of the number of the PDAC/rGO layers; (b) response time as the function of the number of the PDAC/rGO layers; (c) recovery time as the function of the number of the PDAC/rGO layers; (d) Long-term stability curves for the temperature measurements using PDAC/rGO-8L sensor.

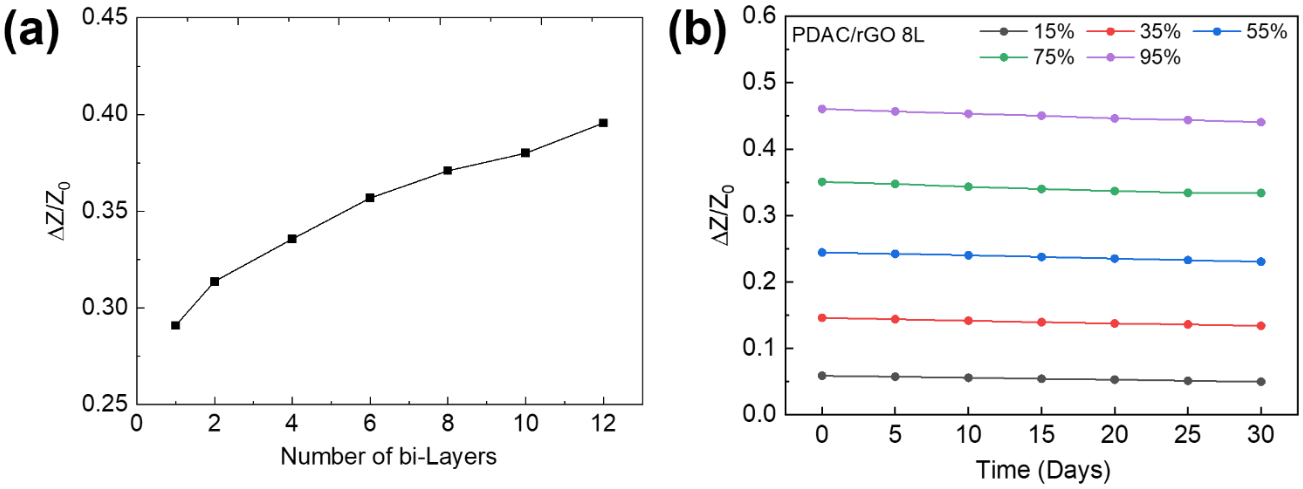

Figure S5. (a) Sensitivity of the response as response curve of different layers for the relative humidity measurements; (b) Long-term stability curves for the relative humidity measurements using PDAC/rGO-8L sensor.

Supplementary Tables:

Table S1. Information of the detected real samples.

Sample	Type	Storage temp.	Storage time
Wine	Rot Tempranillo 2015 Trocken, original liquid (Alcohol: 10.5%)	Room temperature	Newly opened
Coffee	Saturated brewed solution (0.2020 g/ml)	Room temperature	Newly opened
Fresh Beef	Ground meat (10 g)	8 °C	2 days
Spoiled Beef	Ground meat (10 g)	8 °C	10 days
Fresh Pork	Ground meat (10 g)	8 °C	2 days
Spoiled Pork	Ground meat (10 g)	8 °C	10 days
